# Evaluation of three-dimensional SonoAVC ultrasound for antral follicle count in infertile women: its agreement with conventional two-dimensional ultrasound and serum levels of anti-Müllerian hormone

**DOI:** 10.1186/s12958-017-0314-x

**Published:** 2017-12-16

**Authors:** P. A. Peres Fagundes, R. Chapon, P. R. Olsen, A. K. Schuster, M. M. C. Mattia, J. S. Cunha-Filho

**Affiliations:** 1 INSEMINE, Human Reproductive Center, Avenida Dr. Nilo Peçanha 2825, Porto Alegre, 91330-001 Brazil; 2 UFRGS, Universidade Federal do Rio Grande do Sul, Hospital de Clínicas de Porto Alegre, Rua Ramiro Barcelos 2350, Porto Alegre, 90035-903 Brazil; 3 PPGGO, Graduate Program in Health Sciences: Gynecology and Obstetrics, Faculty of Medicine, UFRGS, Rua Ramiro Barcelos, 2400, 2° andar, Porto Alegre, 90035-003 Brazil; 4 UFRGS, Universidade Federal do Rio Grande do Sul, Department of Gynecology and Obstetrics, Porto Alegre Clinical Hospital, Rua Ramiro Barcelos 2350, Porto Alegre, RS 90035-903 Brazil

**Keywords:** Anti-Müllerian hormone, Antral follicle count, Infertility, Transvaginal ultrasound, 3D SonoAVC

## Abstract

**Background:**

Several studies have reported a correlation between antral follicle count by conventional 2D transvaginal sonography and serum anti-Müllerian hormone levels. However, few studies have investigated the effectiveness of 3D SonoAVC transvaginal ultrasound technology, particularly in infertile women. Therefore, this study aims to evaluate the usefulness of three-dimensional (3D) SonoAVC transvaginal ultrasound technology for antral follicle count and its correlation to conventional two-dimensional (2D) transvaginal ultrasound and serum levels of anti-Müllerian hormone in infertile women.

**Methods:**

This cross-sectional study included 42 infertile women with age lower than 40 years that underwent treatment at a private fertility clinic between June and December 2015. Patient data included age, body mass index and cause of infertility. On cycle day 3 the following hormone levels were measured: serum levels of anti-Müllerian hormone, follicle-stimulating hormone, cancer antigen 125, prolactin, thyroid-stimulating hormone and oestradiol; the number of antral follicles was counted as well. The scanning were performed through 2D and 3D technology transvaginal ultrasound.

**Results:**

Using a Bland-Altman test we demonstrated that both technologies are quite equivalent. However, antral follicle count is higher using 3D ultrasound technology compared to 2D technology (*p* < 0.001; Wilcoxon test), this finding is mainly remarkable in ovaries with more than 20 antral follicles. Moreover, the mean time required for manual 2D ultrasound and 3D SonoAVC measurements were 275 ± 109 and 103 ± 57 s, respectively (*p* < 0.001). Serum AMH concentration correlated to the total number of early antral follicles (correlation coefficients = 0.678 and 0.612; *p* < 0.001 by 2D ultrasound and 3D SonoAVC, respectively; Spearman’s correlation test).

**Conclusions:**

Antral follicle count is better estimated using 3D ultrasound compared to 2D technology. A great advantage of 3D SonoAVC was less time required for an examination and the visual advantage when it need to count more than 20 follicles.

**Trial registration:**

CAAE: 35141114.4.0000.5327. Registered 10 June 2015.

## Background

One of the biggest challenges in the management of infertile patients is the loss of reproductive potential with increasing age. The postponement of pregnancy in the modern world is due to several factors, including the pursuit of careers by women, widespread use of contraception, and the development of assisted reproduction techniques. Maternal age is a major factor for decrease in fertility and increase in abortion rates [1–3]. Decreased ovarian reserve with the loss of oocyte quality and depletion of the number of available oocytes with increased age is reflected in the decline in oocyte recovery rate and the decrease in embryo quality and pregnancy rates by in vitro fertilization [1, 2]. The use of follicle - stimulating hormone (FSH), oestradiol (E2), inhibin B, and, more recently, anti-Müllerian hormone (AMH) to evaluate reproductive potential have been described as good markers [4].

Antral follicle count (AFC) has been assessed by two-dimensional (2D) transvaginal ultrasound for several years with good resolution and accuracy. Some authors evaluated the applicability of three-dimensional (3D) technology in comparison with conventional 2D ultrasound to assess the accuracy of this new image management software and did not find any advantage [5]. Furthermore, recently, some authors validated a new mathematical tool to predict ovarian age, using some hormonal and ultrasonographic parameters. Using multiplanar and inversion mode 3D, they showed that AFC is a reliable marker for ovarian reserve, but did not compared to 2D; in addition, the sample was composed mainly by fertile patients [6].

Although few studies had evaluated this new technology in infertile women, there is a need for consensus on a parameter of follicle diameter that represents ovarian functional’s reserve and the usefulness of this emerging technology (3D) compared to 2D. There is also the need to analyze these two different technologies along with serum AMH levels to evaluate ovarian reserve. In this context, it is imperative to evaluate and validate new imaging tools, such as 3D SonoAVC, to explore its potential and define its liabilities.

Therefore, the aim of this study was to identify the agreement between AFCs measured by transvaginal ultrasound (2D and 3D SonoAVC) and serum AMH levels in infertile women.

## Methods

### Study population and design

The Strengthening the Reporting of Observational Studies in Epidemiology guidelines for observational studies was employed in this cross-sectional study of 42 infertile women who underwent treatment at the Department of Obstetrics and Gynecology of Hospital de Clínicas de Porto Alegre or the Insemine Center for Human Reproduction (Porto Alegre, Brazil).

The inclusion criteria were body mass index <30 kg/m^2^, age between 25 and 40 years old, presence of both ovaries, not currently on hormone therapy and no history of chemo and/or radiotherapy. Carrier infertility, which is characterized as the inability to achieve conception or bring a conception to term after a year or more of regular sexual intercourse without contraceptive protection, was also an inclusion criteria.

Exclusion criteria were follicle diameter > 10 mm in both ovaries, ovarian cysts ≥3.0 cm, the presence of endometrioma, injury and/or ovarian masses, the presence of a corpus luteum and/or hemorrhagic cyst body and a history of oophorectomy and/or partial ovarian cystectomy.

### Study protocol

Patients were evaluated at the beginning of the menstrual cycle (days 3–4) by transvaginal ultrasound at the Insemine Center for Human Reproduction using the Voluson E8 Expert ultrasound system (GE Medical Systems, Chicago, IL, USA). The ultrasounds techniques’ data were obtained by the same researcher and were performed in the same day with twenty minutes of interval between both of them. Blood samples were collected for hormonal measurements**.**

### Hormonal measurements

Serum AMH levels were determined using an ultrasensitive enzyme-linked immunosorbent assay (sensitivity, <0.02–10.4 ng/mL; Beckman Coulter, Inc., Brea, CA, USA).

Serum levels of E2 and FSH (Siemens Om-MA Immulite 2000, Munich, Germany) were determined using a chemiluminescence immunoassay. The range of detection of the immunoassay were 10.7 ng/ml–3000 pg/ml for E2 and 0.3–200 mUI/ml for FSH.

### Transvaginal ultrasound

All patients were examined by the same researcher and had both ovaries analyzed. The ultrasounds’ techniques were performed at the beginning of the menstrual cycle using the Voluson E8 Expert ultrasound system (GE Medical Systems, Chicago, IL, USA) equipped with a volumetric transvaginal transducer to count the number of antral follicles and determine the volume of each antral follicle. Ovarian volume was calculated based on diameter using the formula *length × width × depth × 0.523*.

Ultrasound’s settings for data acquisition were standardized for all subjects as follows: transducer frequency, 7.5 MHz; gain, −5; enhance, 2; speckle reduction image, 2; reject level, 25; and harmonic, high.

### Measurement 1: 2D ultrasound image

**Fig. 1:**
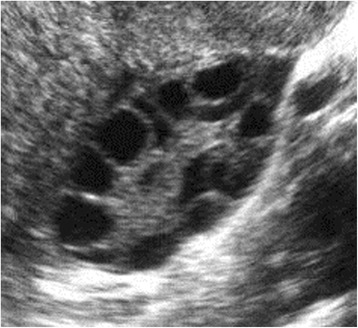
2D ultrasound image to determine antral follicular count. Legend: Ultrasound image by 2D conventional technique that allows the observer to count the number of antral follicles as well as determine its size and volume by *length × width × depth × 0.523*

The number of follicles in two planes, longitudinal and transverse, was determined and the average diameter was calculated. The antral follicle size was determined by two perpendicular diameters (Fig. 1).

### Measurement 2: 3D SonoAVC

**Fig. 2:**
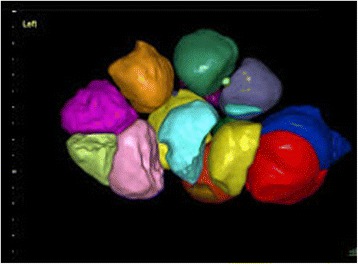
3D ultrasound’s image to determine antral follicle count. Legend: Ultrasound image by 3D SonoAVC’s technique that allows counting the number of antral follicles by individual colors determined by the software. Their volume can also be determined based on the anechoic intrafollicular areas in the scanner that is automatically calculated

The number of follicles in 3D was measured using the software, and the volume of each follicle was automatically calculated based on the anechoic intrafollicular areas in the scanner (Fig. 2). The device was set to analyze only the region of interest with no extra-ovarian structures. The follicular images were displayed with the respective measurements and volumes. Post-processing of the images was manually performed when needed, such as AFCs that were not included in the automatic analysis and the exclusion of adjacent structures. The time spent to perform the follicle count by 2D and 3D SonoAVC were compared.

The same investigator performed all ultrasounds without knowledge of previous 2D or 3D results.

### Statistical analysis

Statistical analysis was performed using SPSS version 22.0 software (IBM-SPSS, Inc., Chicago, IL, USA). The distribution of the variables were analysed through Shapiro-Wilk distribution test. Data that presented parametric distribution were described as mean ± standard deviation (SD), and those with non-parametric distribution were described as median [percentile 25-percentile 75]. Paired t-test or Wilcoxon’s signed-rank test were performed according to the variables, parametric or non-parametric, these tests were performed due to sample’s characteristics; they are dependent observations. For establishing agreement between the two ultrasound methods, Bland-Altman analysis was used [7]. Statistical significance were considered when *p* value <0.05.

An institutional ethics committee approved this research project (IRB equivalent, # CAAE: 35,141,114.4.0000.5327).

## Results

The results regarding the women that underwent the study are presented in the Table 1. The causes of infertility found in this study were sterility without apparent cause (*n* = 8), anatomical factors (*n* = 11), hormonal factors (*n* = 1), endometriosis (*n* = 11), polycystic ovarian syndrome (PCOS; *n* = 6), ovarian factors (*n* = 3) and male factors (2). Considering that 42 women represent 100% that were studied, the causes of infertility represent 19.0%, 26.2%, 2.4%, 26.2%, 14.3%, 7.1% and 4.8%, respectively.

**Table 1:** Characterization of women that underwent the study

Variables	Results	*p* value^*^
Age (years)	33.00[29.75–37.0]	0.049
Body mass index (kg/m^2^)	24.21 ± 2.84	0.48
Anti-Müllerian Hormone level (ng/mL)	2.15[1.03–3.33]	0.0001
Follicle Stimulating Hormone level (mIU/mL)	8.35[6.50–9.05]	0.0001
Oestradiol level (pg/mL)	45.94 ± 16.81	0.914

Legend: Characterization of the women that participated in the study. ^*^ *p* value regarding Shapiro-Wilk for variables’ distribution, the ones with non-parametric distribution are presented as median[P25-P75] and those with parametric distribution are presented as mean ± SD

**Fig. 3:**
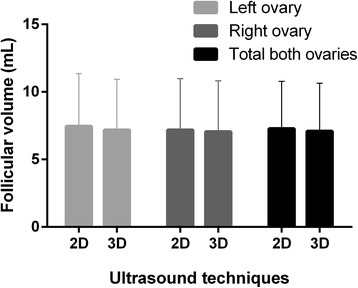
Comparison between 2D and 3D ultrasounds’ techniques regarding follicular volume of left, right and both ovaries. Legend: Analysis of follicular volume in left, right and both ovaries comparing two ultrasounds’ techniques. Left ovary presented mean of 7.46 ± 3.90 and 7.18 ± 3.75 when comparing 2D and 3D ultrasounds’ techniques, respectively. Right ovary presented mean of 7.18 ± 3.80 for 2D ultrasound and 7.06 ± 3.76 for 3D ultrasound. Total follicular volume was obtained through the sum of left and right volume, for 2D and 3D ultrasounds’ techniques, the mean was 7.28 ± 3.50 and 7.10 ± 3.54, respectively. Results are presented as mean bars and standard deviation error bars. Statistical analysis performed through paired t-test has demonstrated no difference between the analysis (*p* ≥ 0.05 for the analysis of left, right and both ovaries)

**Fig. 4:**
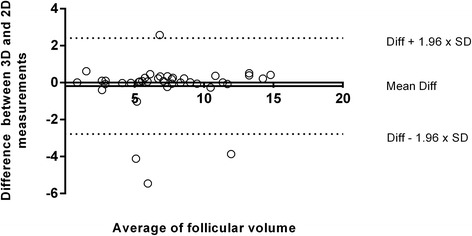
Bland-Altman plot comparing agreement between two ultrasounds’ techniques regarding follicular volume. Legend: Agreement between 2D and 3D ultrasounds’ techniques regarding the mean of follicular volume. The lower (−2.78) and upper (2.41) limits of agreements are represented as dotted lines, the formula to obtain them are found in the right side of the image (SD stands for Standard Deviation); the mean difference (−0.18) is represented as a continuous line. Statistical analysis performed through Bland-Altman (*p* ≥ 0.05; paired t-test)

The same researcher performed both ultrasounds’ techniques, therefore intra-observer correlation was calculated at 0.68 (0.942–0.983) through Spearman’s correlation test. Measurements from the left, right and both ovaries were considered to perform volume analysis. The mean volume of left ovary follicles was 7.46 ± 3.90 for 2D US and 7.18 ± 3.75 for 3D. For the right ovary follicles volume, the mean was 7.18 ± 3.80 and 7.06 and 3.76 for 2D and 3D ultrasounds, respectively. Considering the total volume (left and right ovaries), the mean for 2D US was 7.28 ± 3.50 and the mean for 3D US was 7.10 ± 3.54 (Fig. 3). When comparing the two ultrasounds’ techniques and the follicular volume, there is no statistical difference (*p* ≥ 0.05; *p* ≥ 0.05; *p* ≥ 0.05 for left, right and both ovaries, respectively) (Fig. 4).

**Fig. 5:**
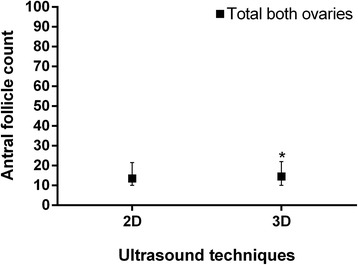
Comparison between two ultrasounds’ techniques regarding left, right and both ovaries antral follicle count. Legend: Antral follicle count performed in both ovaries comparing two ultrasounds’ techniques. The median obtained for 2D and 3D ultrasounds were 13,50[10,00–21,50] and 14,50[10,00–22,00], respectively. Results are presented as median bars and 25–75 percentile error bars. Box signed with (*) represent the statistical significance *p* < 0.001 for total both ovaries analysis, performed through Wilcoxon’s test

**Fig. 6:**
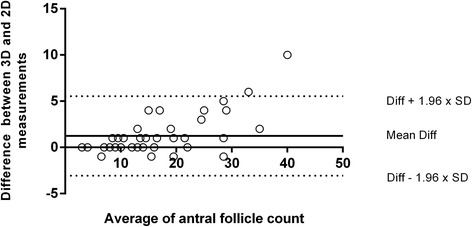
Bland-Altman plot comparing agreement between two ultrasounds’ techniques regarding antral follicle count. Legend: Agreement between 2D and 3D ultrasounds’ techniques regarding the mean of antral follicle count. The lower (−3.06) and upper (5.54) limits of agreements are represented as dotted lines, the formula to obtain them are found in the right side of the image (SD stands for Standard Deviation); The mean difference (2.19) is represented as a continuous line. Statistical analysis performed through Bland-Altman (*p* < 0.001; Wilcoxon’s test)

When comparing both ultrasounds’ techniques regarding AFC, there was statistical difference in left and right ovaries. This data characteristic can also be verified when comparing 2D and 3D techniques and AFC regarding the total AFC, which is represented as mean from both ovaries. The median of total AFC was 13.50 with 10.00 and 21.25 as percentile 25 and 75, respectively for 2D ultrasound. Considering 3D technique, the median number of total antral follicle count was 14.50 with 10.00 and 22.00 as 25 and 75 percentiles respectively, showing statistical difference between both ultrasounds’ techniques. According to Wilcoxon’s signed-rank test, the 3D ultrasound AFC was significantly higher than 2D ultrasound (*p* < 0.001) (Fig. 5). The agreement between both ultrasounds’ techniques regarding AFCs can be found in Fig. 6. The mean time taken to perform manual 2D ultrasound was significantly longer than that taken to perform 3D SonoAVC (275 ± 109 and 103 ± 57 s, respectively; *p* < 0.05; Mann Whitney’s test).

The results presented no correlation between s-AMH levels and BMI (*p* ≥ 0.05). The absence of correlation was observed between AFC and BMI comparing 2D and 3D techniques (*p* ≥ 0.05, for 2D and 3D US; Spearman’s correlation test).

Serum AMH concentration correlated to the total number of early antral follicles (correlation coefficients = 0.678 and 0.612; *p* < 0.001 by 2D ultrasound and 3D SonoAVC, respectively; Spearman’s correlation test).

Ovarian volume correlated to serum AMH concentration (correlation coefficient = 0.558; *p* < 0.001 for 2D US and correlation coefficient = 0.569; *p* < 0.001 for 3D US). Considering only follicles with less than 6 mm, they correlate to s-AMH levels (correlation coefficients = 0.537 and 0.510; *p* < 0.001 for 2D and 3D ultrasounds).

## Discussion

The aim of this study was to identify the agreement between AFCs through two ultrasounds’ techniques in infertile women. This study evaluated the results obtained by 2D conventional ultrasound and 3D SonoAVC ultrasound performed in 42 infertile women.

The study concluded that 3D technology can measure more follicles than 2D, this is particularly important and relevant in ovaries with more than 20 follicles. The better estimation of AFCs by 3D ultrasound was also detected by others investigators, regarding a different group of patients like obese PCOS patients [8]. Weenen et al. also demonstrated that 3D technology was more accurate than 2D when ovaries were submitted to histological confirmation [9].

Our findings are important and opens a new discussion concerning the usefulness of this new technology. We suggest a clinical trial to study ovarian hyperstimulation syndrome (OHSS) and the 3DSonoAVC technique. The 3D ultrasound could be implemented even during infertility investigation, as it is the first step for planning treatment. The fact that AFC was higher using 3D ultrasound did not invalidate 2D technology, both were well correlated to serum AMH levels. However, in high risk patients, such as patients that present PCOS, we should rethink the role of 2D. The positive correlation of AFC to serum AMH and a negative correlation to age observed in our study agree with those of previous studies.

There are both advantages and disadvantages of 3D SonoAVC technology when compared with 2D ultrasound. In traditional 2D follicle count, it is harder to correctly determine the number of follicles when they are presented in a large number, which could lead to over or underestimate follicles count, as described in previous studies [10, 11]. In 3D SonoAVC technology, AFC is performed automatically in a few seconds; the program recognizes anechoic areas of the follicles. The follicular diameter is plotted on three axes (x, y and z) and the follicular volume is calculated automatically, which saves considerable time in routine patient evaluation and increases the quality of the measures. However, these same measures can be performed by conventional 2D ultrasound. The downside of this new 3D SonoAVC technology is the equipment’s higher cost and the need for specialized training for correctly capture 3D images from the ovaries.

Furthermore, in 3D SonoAVC, the ovary must be appropriately placed in the region of interest to prevent the addition of vessels and/or other extraovarian pelvic structures, such as iliac vessels, hydrosalpinx, and Morgagni hydatids. This structures could affect the accuracy and result in under or overestimation of AFC, depending on the experience of the operator and appropriate interpretation of the obtained images. Nevertheless, the automatic image capture process must be evaluated and repeated if considered unsatisfactory [12].Previous studies have reported a good correlation between the results obtained by inter and intra-observer methods [13]. In this study, the same researcher, using the same technique and settings, captured all images and the intra-observer correlation was calculated.

A benefit of 3D SonoAVC observed in this study was the ease of quickly obtaining reproducible measurements, particularly for small follicles. However, after image capture, the examiner must assess image quality and verify if the automatic count matches expectations. This software’s characteristics can improve performance and workflow in fertility clinics where the number of follicles to be measured is sometimes numerous.

Another interesting possibility of this new technology is the ability to store images in a 3D virtual block for further detailed studies in case of special interest or to create a research database. The future prospects of research in this field are promising with the advent of new equipment and image management’s technologies to enhance the knowledge of ovarian folliculogenesis, particularly for AFCs when the follicle diameter is less than 2 mm.

The main limitation about our results was the fact that we cannot certify the accuracy of both methods using histology, Deb et al., 2010, already discussed this. Also, the exclusion of obese patients (BMI > 30 kg/m2) could be seen as conservative bias in favor to 2D technology. We conducted AFCs in the initial menstrual cycle’s phase to minimize the effect of intracycle variation of follicular diameters and avoid inclusion of ovarian cysts or a pre-existing corpus luteum, which were considered in the exclusion criteria in this population. The measurements performed by the same researcher in both ultrasounds’ techniques were obtained with twenty minutes of interval between them. Although we tried to minimize the bias, this observation could be assessed as one weakness of the study.

## Conclusions

In conclusion, the results of this study suggest that 3D SonoAVC can estimate AFCs better when compared to 2D ultrasound, mainly because it can capture images of ovaries with more than 20 follicles, resulting in a more reliable counting and was found to be faster than 2D for AFC. In addition, AFCs by both technologies presented a positive correlation to serum AMH levels.

## Abbreviations

2D: Two-dimensional
3D: Three-dimensional
AFC: Antral follicle count
AMH: Anti-Müllerian hormone
CA-125: Cancer antigen 125
E2: Oestradiol
FSH: Follicle-stimulating hormone
OHSS: Ovarian hyperstimulation syndrome
PCOS: Polycystic ovarian syndrome
SD: Standard deviation
TSH: Thyroid-stimulating hormone

## References

[1] La Marca A, Muttukrishna S, Cunha-Filho JS (2011). Age-related normograms of serum antimüllerian hormone levels in a population of infertile women: a multicenter study. Fertil Steril.

[2] de Vet A, Laven JSE, de Jong FH, Themmen APN, Fauser BC (2002). Anti-müllerian hormone serum levels: a putative marker for ovarian aging. Fertil Steril.

[3] Bonilla-Musoles F, Castillo JC, Caballero O (2012). Predicting ovarian reserve and reproductive outcome using antimüllerian hormone (AMH) and antral follicle count (AFC) in patients with previous assisted reproduction technique (ART) failure. Clin Exp Obstet Gynecol.

[4] Fanchin R, Schonauer LM, Righini C, Guibourdenche J, Frydman R, Taieb J (2003). Serum anti-Müllerian hormone is more strongly related to ovarian folicular status than serum innibin B, oestradiol, FSH and LH on day 3. Hum Reprod.

[5] Jayaprakasan K, Hilwah N, Kendall NR (2007). Does 3D ultrasound offer any advantage in the pretreatment assessment of ovarian reserve and prediction of outcome after assisted reproduction treatment. Hum Reprod.

[6] Venturella R, Lico D, Sarica A (2015). OvAge: a new methodology to quantify ovarian reserve combining clinical, biochemical and 3D-ultrasonographic parameters. J Ovarian Res.

[7] Bland JM, Altman DG (1986). Statistical methods for assessing agreement between two methods of clinical measurement. Lancet.

[8] Nylander M (2017). Ovarian morphology in polycystic ovarian syndrome: estimates from 2D and 3D ultrasound and magnetic resonance imaging and their correlation to anti-Müllerian hormone. Acta Radiol.

[9] Weenen C (2004). Anti-Müllerian hormone expression pattern in human ovary: potential implications for initial and cyclic follicle recruitment. Mol Hum Reprod.

[10] Deb SR, Campbell BK, Cleves J, Porter N, Winter B, Raine-Fenning N (2010). Quantitative analysis of number and size of antral follicles: a comparison of real-time two-dimensional and automated three-dimensional ultrasound. Ultrasound Obstet Gynecol.

[11] Raine-Fenning N, Jayaprakasan K, Clewes J (2007). Automated follicle tracking facilitates standardization and may improve work flow. Ultrasound Obstet Gynecol.

[12] Kyei-Mensah A, Zaidi J, Pittrof R, Shaker A, Campbell S, Tan SL (1996). Transvaginal three dimensional ultrasound: accuracy of follicular volume measurements. Fertil Steril.

[13] Deb S, Jayaprakasan K, Campbell BK, Clewes JS, Johnson IR, Raine-Fenning NJ (2009). Intraobserver and interobserver reliability of automated antral follicle counts made using three-dimensional ultrasound and SonoAVC. Ultrasound Obstet Gynecol.

